# RETRACTION: β-Elemene Promotes Apoptosis Induced by Hyperthermia via Inhibiting HSP70

**DOI:** 10.1155/dim/9872193

**Published:** 2025-11-10

**Authors:** Disease Markers

RETRACTION: C. Zhang, S. Li, and Z. Zhao, “β-Elemene Promotes Apoptosis Induced by Hyperthermia via Inhibiting HSP70,” *Disease Markers*, no. 2022 (2022), https://doi.org/10.1155/2022/7313026.

The above article, published online on 19 July 2022 in Wiley Online Library (wileyonlinelibrary.com), has been retracted by John Wiley & Sons Ltd.

The retraction has been agreed following an investigation of the concerns flagged by *Mycosphaerella arachidis* and *Hoya Camphorifolia* on PubPeer [1], which identified multiple instances of figure duplication.

Specifically:

- Figure 2b: The image of the cell colony grown at 37 °C is identical to the cells shown in Figure 2d (top left) of [2], where they represent a different cell culture. Concerns have also been identified regarding the similarity of the colony grown under 42 °C to Figure 4b (top right).
- Figure 2c: Multiple images share unexpected similarities with the tumor cells in Figure 2c of [3], which were cultured under different experimental conditions.
- Figure 5a: Similar features have been detected between multiple panels of the figure, even though the Transwell assays they represented were performed under different conditions.

As a result of the investigation, the data and conclusions of this article are considered unreliable.

The authors have been informed of this retraction but did not provide a response.
